# Poly[tris­(μ-2-amino­benzene-1,4-dicarboxyl­ato)tetra­kis­(*N*,*N*-dimethyl­formamide)­diyttrium(III)]

**DOI:** 10.1107/S1600536810050555

**Published:** 2010-12-08

**Authors:** Mathivathani Kandiah, David Stephen Wragg, Mats Tilset, Karl Petter Lillerud

**Affiliations:** a inGAP Centre for Research Based Innovation, Department of Chemistry, University of Oslo, PO Box 1033 Blindern, 0315 Oslo, Norway

## Abstract

The asymmetric unit of the title coordination polymer, [Y_2_(C_8_H_5_NO_4_)_3_(C_3_H_7_NO)_4_]_*n*_, contains one Y^3+^ ion, three half-mol­ecules of the 2-amino­benzene-1,4-dicarboxyl­ate (abz) dianion and two *O*-bonded *N*,*N*-dimethyl­formamide (DMF) mol­ecules. Each abz half-mol­ecule is completed by crystallographic inversion symmetry and its –NH_2_ group is disordered in each case [relative occupancies within the asymmetric unit = 0.462 (18):0.538 (18), 0.93 (2):0.07 (2) and 0.828 (16):0.172 (16)]. The combination of disorder and crystal symmetry means that each of the four C—H atoms of the benzene ring of each of the dianions bears a statistical fraction of an –NH_2_ group. The coordination geometry of the yttrium ion is a fairly regular YO_8_ square anti­prism arising from its coordination by two DMF mol­ecules, four monodentate abz dianions and one *O*,*O*-bidentate abz dianion. The polymeric building unit is a dimeric paddle-wheel with two metal ions linked by four bridging abz dianions. Further bridging linkages connect the dimers into a three-dimensional framework containing voids in which highly disordered DMF mol­ecules are presumed to reside.

## Related literature

For a related structure containing a similar paddle-wheel motif, see: Braun *et al.* (2001 ▶). 
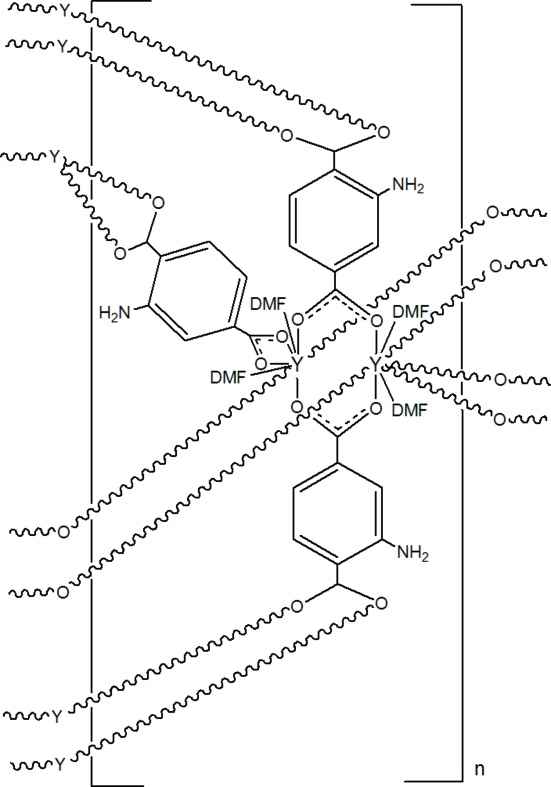


## Experimental

### 

#### Crystal data

[Y_2_(C_8_H_5_NO_4_)_3_(C_3_H_7_NO)_4_]
*M* *_r_* = 1007.59
Triclinic, 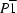

*a* = 10.525 (3) Å
*b* = 11.034 (3) Å
*c* = 12.855 (3) Å
α = 99.359 (3)°
β = 111.301 (3)°
γ = 101.265 (2)°
*V* = 1318.8 (6) Å^3^
*Z* = 1
Mo *K*α radiation
μ = 2.25 mm^−1^
*T* = 150 K
0.25 × 0.22 × 0.05 mm

#### Data collection

Bruker APEX CCD diffractometer
Absorption correction: multi-scan (*SADABS*; Sheldrick, 2004 ▶) *T*_min_ = 0.575, *T*_max_ = 0.894
7363 measured reflections
2345 independent reflections
2111 reflections with *I* > 2σ(*I*)
*R*_int_ = 0.027
θ_max_ = 19.7°

#### Refinement

*R*[*F* ^2^ > 2σ(*F* ^2^)] = 0.043
*wR*(*F* ^2^) = 0.123
*S* = 1.11
2345 reflections
298 parameters
46 restraints
H-atom parameters constrained
Δρ_max_ = 0.71 e Å^−3^
Δρ_min_ = −0.41 e Å^−3^

### 

Data collection: *SMART* (Bruker, 2001 ▶); cell refinement: *SAINT* (Bruker, 2001 ▶); data reduction: *SAINT*; program(s) used to solve structure: *SHELXS97* (Sheldrick, 2008 ▶); program(s) used to refine structure: *SHELXL97* (Sheldrick, 2008 ▶); molecular graphics: *DIAMOND* (Crystal Impact, 2004) ▶; software used to prepare material for publication: *PLATON* (Spek, 2009 ▶).

## Supplementary Material

Crystal structure: contains datablocks I, global. DOI: 10.1107/S1600536810050555/hb5707sup1.cif

Structure factors: contains datablocks I. DOI: 10.1107/S1600536810050555/hb5707Isup2.hkl

Additional supplementary materials:  crystallographic information; 3D view; checkCIF report

## Figures and Tables

**Table 1:** Selected bond lengths (Å)

Y1—O3	2.252 (5)
Y1—O1	2.311 (4)
Y1—O5	2.322 (5)
Y1—O7	2.335 (4)
Y1—O8	2.358 (6)
Y1—O2	2.361 (6)
Y1—O6	2.409 (4)
Y1—O4	2.416 (4)

**Fig. 1.**
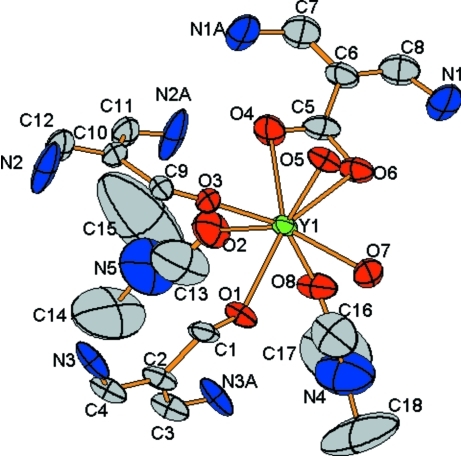
ORTEP view of the asymmetric unit of (I) with thermal ellipsoids at 50% probability. Hydrogen atoms are omitted for clarity.

**Fig. 2.**
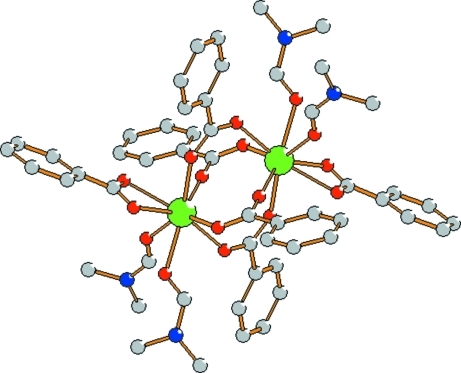
The paddle wheel unit of the MOF structure of (I).

**Fig. 3.**
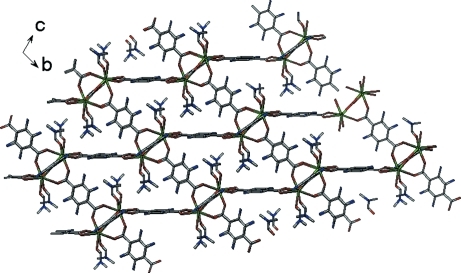
Packing diagram of (I) viewed along the a-axis.

**Fig. 4.**
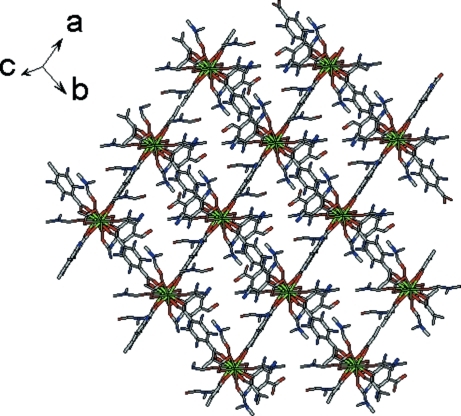
Packing diagram of (I) viewed along the axis of the paddle wheel unit.

## supplementary crystallographic information

### Comment

The title compound (I) is a metal-organic framework (MOF) which is a weak scatterer of X-rays and data were collected from a very small crystal. These were sufficient for refinement of the framework structure but not the disordered solvent. The asymmetric unit (Fig. 1) of the title compound consists of an yttrium atom coordinated by 8 oxygen atoms, 6 from 2-amino-1,4-benzenedicarboxylic acid (BDC-NH_2_) moieties and 2 from coordinated dimethylformamide solvent. The inorganic cornerstone of the MOF is a paddle wheel type unit comprising two yttrium atoms linked by four bridging Y-BDC-NH_2_ molecules (Fig. 2). Such units are well known in transition metal MOF structures (Braun, 2001). The paddle wheel carboxylates clearly show one long and one short C—O bond indicating single and double bond character. The single bonded, charge carrying oxygen of the carboxylate group has a shorter Y—O bond distance as would be expected. The axles of the paddle wheel are connected to one BDC-NH_2_ and 2 DMF molecules at each end.

The BDC-NH_2_ linkers at the ends of the paddle wheel are offset in a *trans*-type conformation and link the units in chains which, when the structure is viewed along the *a*-axis (Fig. 3), bisect the angle of the *b* and *c* axes. When we view the structure along the axis of the paddle wheel unit (Fig. 4) we see that the bridging BDC-NH_2_ molecules also link to further units in chains parallel to the *a* and *b*-axes producing a three-dinemsional network. The amino groups are disordered over all four possible positions of the benzene rings of the BDC-NH_2_ linker molecules, except in the case of the ring described by C10, C11 and C12, in which the NH_2_ was found to be localized on C12. All of the disordered C—N bonds were restrained to have the same bond distance.

1 molecule of DMF solvent per ASU was located in the void space of the MOF using difference Fourier maps but the resulting model gave a poor refinement. The program Squeeze from the *PLATON* suite (Spek, 2009 ) was used to remove residual electron density from the solvent accessible voids giving a chemically sensible structure and acceptable refinemnt statistics. The squeeze calculation suggests voids containing 90 electrons or 2.25 DMF molecules in each unit cell. This is in reasonable agreement with the disordered solvent observed in the difference Fourier map.

### Experimental

The Title complex Y-BDC-NH_2_ was prepared by dissolving Y(NO_3_)_3_.6H_2_O (0.383 g, 1 mmol) and 2-amino 1,4-benzenedicarboxylic acid (H_2_N—H_2_BDC) (0.181 g, 1 mmol) in *N*,*N*`-dimethylformamide (DMF) (20 ml) at room temperature in a test tube. The mixture thus obtained was placed in a pre-heated oven at 80°C for 24 hrs. Colourless plates of (I) were selected directly from the mother liquor as prepared and mounted on cryoloops.

### Refinement

Most of the non hydrogen atoms positions were obtained from the direct methods solution and the remainder (mainly carbon atoms) were located using difference Fourier maps during refinement. Hydrogen atoms were placed in ideal positions and refined with a riding model.

### Crystal data

**Table:**

[Y_2_(C_8_H_5_NO_4_)_3_(C_3_H_7_NO)_4_]	*Z* = 1
*M**_r_* = 1007.59	*F*(000) = 505
Triclinic, *P*1	*D*_x_ = 1.257 Mg m^−^^3^
Hall symbol: -P 1	Mo *K*α radiation, λ = 0.71073 Å
*a* = 10.525 (3) Å	Cell parameters from 3097 reflections
*b* = 11.034 (3) Å	θ = 2.4–28.2°
*c* = 12.855 (3) Å	µ = 2.25 mm^−^^1^
α = 99.359 (3)°	*T* = 150 K
β = 111.301 (3)°	Plate, colourless
γ = 101.265 (2)°	0.25 × 0.22 × 0.05 mm
*V* = 1318.8 (6) Å^3^	

### Data collection

**Table:**

Bruker APEX CCD diffractometer	2345 independent reflections
Radiation source: sealed tube	2111 reflections with *I* > 2σ(*I*)
graphite	*R*_int_ = 0.027
phi and ω scans	θ_max_ = 19.7°, θ_min_ = 1.8°
Absorption correction: multi-scan (*SADABS*; Sheldrick, 2004)	*h* = −9→9
*T*_min_ = 0.575, *T*_max_ = 0.894	*k* = −10→10
7363 measured reflections	*l* = −12→12

### Refinement

**Table:**

Refinement on *F*^2^	Primary atom site location: structure-invariant direct methods
Least-squares matrix: full	Secondary atom site location: difference Fourier map
*R*[*F*^2^ > 2σ(*F*^2^)] = 0.043	Hydrogen site location: inferred from neighbouring sites
*wR*(*F*^2^) = 0.123	H-atom parameters constrained
*S* = 1.11	*w* = 1/[σ^2^(*F*_o_^2^) + (0.0707*P*)^2^ + 2.6497*P*] where *P* = (*F*_o_^2^ + 2*F*_c_^2^)/3
2345 reflections	(Δ/σ)_max_ = 0.002
298 parameters	Δρ_max_ = 0.71 e Å^−^^3^
46 restraints	Δρ_min_ = −0.41 e Å^−^^3^

### Special details

**Table:**

Geometry. All s.u.'s (except the s.u. in the dihedral angle between two l.s. planes) are estimated using the full covariance matrix. The cell s.u.'s are taken into account individually in the estimation of s.u.'s in distances, angles and torsion angles; correlations between s.u.'s in cell parameters are only used when they are defined by crystal symmetry. An approximate (isotropic) treatment of cell s.u.'s is used for estimating s.u.'s involving l.s. planes.
Refinement. Refinement of *F*^2^ against ALL reflections. The weighted *R*-factor *wR* and goodness of fit *S* are based on *F*^2^, conventional *R*-factors *R* are based on *F*, with *F* set to zero for negative *F*^2^. The threshold expression of *F*^2^ > 2σ(*F*^2^) is used only for calculating *R*-factors(gt) *etc*. and is not relevant to the choice of reflections for refinement. *R*-factors based on *F*^2^ are statistically about twice as large as those based on *F*, and *R*- factors based on ALL data will be even larger.

### Fractional atomic coordinates and isotropic or equivalent isotropic displacement parameters (Å^2^)

**Table:**

	*x*	*y*	*z*	*U*_iso_*/*U*_eq_	Occ. (<1)
Y1	0.60986 (6)	0.54621 (5)	0.67714 (5)	0.0325 (3)	
O1	0.5003 (5)	0.3303 (4)	0.5952 (4)	0.0458 (12)	
O2	0.7957 (6)	0.4535 (6)	0.7557 (5)	0.0777 (17)	
O3	0.6664 (4)	0.5086 (4)	0.5242 (4)	0.0489 (12)	
O4	0.8360 (5)	0.7070 (5)	0.7643 (5)	0.0628 (15)	
O5	0.5711 (5)	0.7170 (4)	0.5974 (4)	0.0429 (11)	
O6	0.6807 (5)	0.7297 (4)	0.8365 (4)	0.0512 (13)	
O7	0.3832 (5)	0.5508 (4)	0.6623 (5)	0.0497 (12)	
O8	0.5809 (6)	0.4663 (5)	0.8289 (5)	0.0664 (16)	
C1	0.5309 (7)	0.7437 (6)	0.5023 (7)	0.0425 (18)	
C2	0.5175 (7)	0.8788 (6)	0.5026 (6)	0.0450 (18)	
C3	0.5023 (8)	0.9231 (7)	0.4060 (6)	0.054 (2)	
H3	0.5042	0.8702	0.3407	0.064*	0.70
C4	0.5155 (8)	0.9569 (6)	0.5979 (6)	0.0504 (19)	
H4	0.5264	0.9278	0.6654	0.060*	0.80
C5	0.8016 (8)	0.7688 (7)	0.8353 (6)	0.0481 (19)	
C6	0.9007 (8)	0.8887 (6)	0.9175 (6)	0.0507 (19)	
C7	1.0407 (9)	0.9239 (8)	0.9287 (7)	0.075 (3)	
H7	1.0708	0.8716	0.8812	0.090*	0.75
C8	0.8620 (8)	0.9631 (8)	0.9897 (7)	0.071 (2)	
H8	0.7680	0.9386	0.9851	0.085*	0.75
C9	0.7040 (8)	0.4813 (6)	0.4410 (8)	0.0452 (18)	
C10	0.8564 (7)	0.4903 (6)	0.4706 (6)	0.0426 (18)	
C11	0.9565 (8)	0.5420 (7)	0.5837 (6)	0.052 (2)	
H11	0.9267	0.5707	0.6424	0.062*	
C12	0.9036 (7)	0.4478 (7)	0.3883 (6)	0.052 (2)	
H12	0.8372	0.4107	0.3106	0.062*	0.50
C13	0.8097 (13)	0.3559 (13)	0.7616 (12)	0.146 (6)	
H13	0.7299	0.2897	0.7062	0.175*	
C14	0.8993 (18)	0.1782 (16)	0.8060 (19)	0.234 (10)	
H14A	0.9844	0.1670	0.8638	0.351*	0.50
H14B	0.8939	0.1452	0.7286	0.351*	0.50
H14C	0.8149	0.1314	0.8132	0.351*	0.50
H14D	0.8111	0.1288	0.7399	0.351*	0.50
H14E	0.9016	0.1505	0.8752	0.351*	0.50
H14F	0.9806	0.1643	0.7905	0.351*	0.50
C15	1.042 (3)	0.393 (2)	0.903 (2)	0.39 (2)	
H15A	1.1003	0.3414	0.9425	0.590*	0.50
H15B	1.0311	0.4561	0.9592	0.590*	0.50
H15C	1.0882	0.4364	0.8598	0.590*	0.50
H15D	1.0461	0.4812	0.8985	0.590*	0.50
H15E	1.1153	0.3666	0.8818	0.590*	0.50
H15F	1.0582	0.3862	0.9812	0.590*	0.50
C16	0.4818 (15)	0.3945 (12)	0.8288 (9)	0.103 (3)	
H16	0.3967	0.3770	0.7615	0.124*	
C17	0.595 (2)	0.3593 (16)	1.0148 (12)	0.195 (8)	
H17A	0.5719	0.3094	1.0652	0.292*	0.50
H17B	0.6263	0.4506	1.0539	0.292*	0.50
H17C	0.6713	0.3351	0.9974	0.292*	0.50
H17D	0.6744	0.4206	1.0125	0.292*	0.50
H17E	0.6200	0.2794	1.0237	0.292*	0.50
H17F	0.5750	0.3949	1.0802	0.292*	0.50
C18	0.354 (2)	0.2376 (18)	0.9021 (16)	0.244 (11)	
H18A	0.3789	0.2133	0.9755	0.366*	0.50
H18B	0.3319	0.1625	0.8395	0.366*	0.50
H18C	0.2707	0.2706	0.8872	0.366*	0.50
H18D	0.2754	0.2176	0.8260	0.366*	0.50
H18E	0.3225	0.2684	0.9620	0.366*	0.50
H18F	0.3836	0.1604	0.9143	0.366*	0.50
N1	0.7183 (19)	0.959 (3)	0.972 (4)	0.137 (12)	0.228 (9)
H1A	0.6474	0.9049	0.9113	0.165*	0.228 (9)
H1B	0.7011	1.0097	1.0227	0.165*	0.228 (9)
N1A	1.101 (3)	0.857 (3)	0.862 (3)	0.137 (12)	0.272 (9)
H1A1	1.0485	0.7865	0.8076	0.165*	0.272 (9)
H1A2	1.1912	0.8867	0.8752	0.165*	0.272 (9)
N2	0.8114 (13)	0.3877 (17)	0.2719 (10)	0.111 (7)	0.50
H2A	0.8456	0.3596	0.2226	0.134*	0.50
H2B	0.7196	0.3783	0.2487	0.134*	0.50
N3	0.5205 (18)	0.9180 (14)	0.7009 (11)	0.079 (6)	0.424 (9)
H3A	0.5124	0.9699	0.7563	0.094*	0.424 (9)
H3B	0.5317	0.8424	0.7082	0.094*	0.424 (9)
N3A	0.499 (10)	0.857 (7)	0.299 (4)	0.079 (6)	0.076 (9)
H3A1	0.5078	0.7784	0.2905	0.094*	0.076 (9)
H3A2	0.4890	0.8945	0.2426	0.094*	0.076 (9)
N4	0.4703 (14)	0.3341 (10)	0.9082 (10)	0.143 (4)	
N5	0.9061 (13)	0.3113 (12)	0.8242 (12)	0.157 (5)	

### Atomic displacement parameters (Å^2^)

**Table:**

	*U*^11^	*U*^22^	*U*^33^	*U*^12^	*U*^13^	*U*^23^
Y1	0.0242 (4)	0.0219 (4)	0.0408 (5)	0.0023 (3)	0.0060 (3)	0.0025 (3)
O1	0.057 (3)	0.025 (3)	0.043 (3)	0.004 (2)	0.013 (2)	0.002 (2)
O2	0.064 (4)	0.065 (4)	0.100 (5)	0.033 (3)	0.019 (3)	0.027 (4)
O3	0.036 (3)	0.035 (3)	0.073 (4)	0.003 (2)	0.029 (3)	−0.002 (2)
O4	0.038 (3)	0.047 (3)	0.072 (4)	−0.003 (2)	0.010 (3)	−0.020 (3)
O5	0.049 (3)	0.026 (3)	0.040 (3)	0.004 (2)	0.007 (2)	0.005 (2)
O6	0.053 (4)	0.035 (3)	0.045 (3)	−0.007 (2)	0.012 (3)	−0.001 (2)
O7	0.029 (3)	0.050 (3)	0.058 (4)	0.006 (2)	0.012 (3)	0.003 (3)
O8	0.073 (4)	0.043 (3)	0.069 (4)	−0.006 (3)	0.026 (3)	0.013 (3)
C1	0.036 (4)	0.023 (4)	0.054 (6)	−0.003 (3)	0.011 (4)	0.005 (4)
C2	0.048 (4)	0.025 (4)	0.042 (5)	−0.002 (3)	0.005 (4)	0.001 (4)
C3	0.075 (5)	0.029 (5)	0.042 (5)	0.005 (4)	0.014 (4)	0.005 (4)
C4	0.074 (5)	0.026 (4)	0.037 (5)	0.005 (4)	0.010 (4)	0.011 (4)
C5	0.034 (5)	0.038 (5)	0.043 (5)	−0.007 (4)	−0.006 (4)	0.006 (4)
C6	0.051 (6)	0.033 (4)	0.050 (5)	−0.004 (4)	0.014 (4)	−0.004 (4)
C7	0.059 (6)	0.061 (6)	0.078 (6)	−0.003 (5)	0.022 (5)	−0.014 (5)
C8	0.050 (5)	0.063 (6)	0.071 (6)	−0.008 (5)	0.012 (5)	0.003 (5)
C9	0.038 (5)	0.029 (4)	0.068 (6)	0.008 (3)	0.025 (5)	0.003 (4)
C10	0.028 (4)	0.038 (4)	0.055 (5)	0.003 (3)	0.018 (5)	−0.001 (4)
C11	0.038 (5)	0.058 (5)	0.052 (6)	0.006 (4)	0.025 (4)	−0.011 (4)
C12	0.025 (5)	0.064 (5)	0.047 (5)	0.004 (4)	0.008 (4)	−0.009 (4)
C13	0.087 (9)	0.096 (10)	0.190 (14)	0.024 (8)	−0.015 (9)	0.038 (10)
C14	0.165 (15)	0.162 (15)	0.39 (3)	0.082 (13)	0.069 (17)	0.181 (18)
C15	0.32 (3)	0.23 (2)	0.36 (3)	0.14 (2)	−0.13 (3)	−0.08 (2)
C16	0.146 (11)	0.102 (9)	0.067 (7)	0.034 (8)	0.045 (7)	0.029 (7)
C17	0.26 (2)	0.192 (16)	0.100 (11)	0.050 (15)	0.037 (13)	0.061 (11)
C18	0.23 (2)	0.26 (2)	0.236 (19)	−0.063 (16)	0.147 (17)	0.100 (17)
N1	0.062 (15)	0.114 (19)	0.18 (3)	−0.036 (13)	0.061 (16)	−0.079 (18)
N1A	0.062 (15)	0.114 (19)	0.18 (3)	−0.036 (13)	0.061 (16)	−0.079 (18)
N2	0.031 (8)	0.196 (18)	0.066 (10)	0.025 (9)	0.006 (8)	−0.035 (11)
N3	0.116 (14)	0.044 (9)	0.069 (11)	0.041 (9)	0.020 (10)	0.019 (8)
N3A	0.116 (14)	0.044 (9)	0.069 (11)	0.041 (9)	0.020 (10)	0.019 (8)
N4	0.192 (12)	0.124 (8)	0.099 (8)	0.000 (8)	0.063 (8)	0.040 (7)
N5	0.121 (9)	0.149 (11)	0.199 (12)	0.089 (8)	0.016 (8)	0.096 (10)

### Geometric parameters (Å, °)

**Table:**

Y1—O3	2.252 (5)	C12—N2	1.411 (11)
Y1—O1	2.311 (4)	C12—H12	0.9500
Y1—O5	2.322 (5)	C13—N5	1.284 (14)
Y1—O7	2.335 (4)	C13—H13	0.9500
Y1—O8	2.358 (6)	C14—N5	1.433 (18)
Y1—O2	2.361 (6)	C14—H14A	0.9800
Y1—O6	2.409 (4)	C14—H14B	0.9800
Y1—O4	2.416 (4)	C14—H14C	0.9800
Y1—C5	2.775 (7)	C14—H14D	0.9800
Y1—C9^i^	3.021 (7)	C14—H14E	0.9800
O1—C1^i^	1.268 (8)	C14—H14F	0.9800
O2—C13	1.126 (13)	C15—N5	1.42 (2)
O3—C9	1.281 (8)	C15—H15A	0.9800
O4—C5	1.251 (9)	C15—H15B	0.9800
O5—C1	1.245 (8)	C15—H15C	0.9800
O6—C5	1.268 (8)	C15—H15D	0.9800
O7—C9^i^	1.252 (8)	C15—H15E	0.9800
O8—C16	1.179 (13)	C15—H15F	0.9800
C1—O1^i^	1.268 (8)	C16—N4	1.335 (13)
C1—C2	1.524 (9)	C16—H16	0.9500
C2—C3	1.375 (9)	C17—N4	1.451 (17)
C2—C4	1.388 (9)	C17—H17A	0.9800
C3—C4^ii^	1.379 (9)	C17—H17B	0.9800
C3—N3A	1.432 (13)	C17—H17C	0.9800
C3—H3	0.9500	C17—H17D	0.9800
C4—C3^ii^	1.379 (9)	C17—H17E	0.9800
C4—N3	1.443 (11)	C17—H17F	0.9800
C4—H4	0.9500	C18—N4	1.427 (16)
C5—C6	1.470 (10)	C18—H18A	0.9800
C6—C8	1.364 (11)	C18—H18B	0.9800
C6—C7	1.395 (11)	C18—H18C	0.9800
C7—C8^iii^	1.414 (11)	C18—H18D	0.9800
C7—N1A	1.436 (12)	C18—H18E	0.9800
C7—H7	0.9500	C18—H18F	0.9800
C8—C7^iii^	1.414 (11)	N1—H1A	0.8800
C8—N1	1.436 (13)	N1—H1B	0.8800
C8—H8	0.9500	N1A—H1A1	0.8800
C9—O7^i^	1.252 (8)	N1A—H1A2	0.8800
C9—C10	1.487 (10)	N2—H2A	0.8800
C9—Y1^i^	3.021 (7)	N2—H2B	0.8800
C10—C12	1.377 (9)	N3—H3A	0.8800
C10—C11	1.390 (9)	N3—H3B	0.8800
C11—C12^iv^	1.359 (9)	N3A—H3A1	0.8800
C11—H11	0.9500	N3A—H3A2	0.8800
C12—C11^iv^	1.359 (9)		
			
O3—Y1—O1	77.48 (15)	H14C—C14—H14D	56.3
O3—Y1—O5	76.70 (16)	N5—C14—H14E	109.5
O1—Y1—O5	128.81 (15)	H14A—C14—H14E	56.3
O3—Y1—O7	123.79 (17)	H14B—C14—H14E	141.1
O1—Y1—O7	82.81 (16)	H14C—C14—H14E	56.3
O5—Y1—O7	76.18 (16)	H14D—C14—H14E	109.5
O3—Y1—O8	145.22 (18)	N5—C14—H14F	109.5
O1—Y1—O8	75.52 (17)	H14A—C14—H14F	56.3
O5—Y1—O8	137.92 (18)	H14B—C14—H14F	56.3
O7—Y1—O8	73.86 (19)	H14C—C14—H14F	141.1
O3—Y1—O2	81.36 (19)	H14D—C14—H14F	109.5
O1—Y1—O2	77.45 (19)	H14E—C14—H14F	109.5
O5—Y1—O2	139.42 (19)	N5—C15—H15A	109.5
O7—Y1—O2	143.6 (2)	N5—C15—H15B	109.5
O8—Y1—O2	71.7 (2)	H15A—C15—H15B	109.5
O3—Y1—O6	133.34 (16)	N5—C15—H15C	109.5
O1—Y1—O6	148.63 (17)	H15A—C15—H15C	109.5
O5—Y1—O6	73.50 (15)	H15B—C15—H15C	109.5
O7—Y1—O6	82.41 (18)	N5—C15—H15D	109.5
O8—Y1—O6	73.84 (17)	H15A—C15—H15D	141.1
O2—Y1—O6	99.00 (19)	H15B—C15—H15D	56.3
O3—Y1—O4	84.05 (18)	H15C—C15—H15D	56.3
O1—Y1—O4	144.15 (17)	N5—C15—H15E	109.5
O5—Y1—O4	74.57 (17)	H15A—C15—H15E	56.3
O7—Y1—O4	132.62 (17)	H15B—C15—H15E	141.1
O8—Y1—O4	105.82 (19)	H15C—C15—H15E	56.3
O2—Y1—O4	69.5 (2)	H15D—C15—H15E	109.5
O6—Y1—O4	53.92 (17)	N5—C15—H15F	109.5
C1^i^—O1—Y1	133.9 (4)	H15A—C15—H15F	56.3
C13—O2—Y1	138.5 (8)	H15B—C15—H15F	56.3
C9—O3—Y1	175.4 (5)	H15C—C15—H15F	141.1
C5—O4—Y1	92.8 (4)	H15D—C15—H15F	109.5
C1—O5—Y1	141.0 (4)	H15E—C15—H15F	109.5
C5—O6—Y1	92.7 (4)	O8—C16—N4	130.2 (12)
C9^i^—O7—Y1	111.1 (4)	O8—C16—H16	114.9
C16—O8—Y1	129.7 (7)	N4—C16—H16	114.9
O5—C1—O1^i^	126.5 (6)	N4—C17—H17A	109.5
O5—C1—C2	117.0 (7)	N4—C17—H17B	109.5
O1^i^—C1—C2	116.5 (7)	H17A—C17—H17B	109.5
C3—C2—C4	119.1 (6)	N4—C17—H17C	109.5
C3—C2—C1	119.9 (7)	H17A—C17—H17C	109.5
C4—C2—C1	120.9 (7)	H17B—C17—H17C	109.5
C2—C3—C4^ii^	121.2 (7)	N4—C17—H17D	109.5
C2—C3—N3A	127 (3)	H17A—C17—H17D	141.1
C4^ii^—C3—N3A	111 (3)	H17B—C17—H17D	56.3
C2—C3—H3	119.4	H17C—C17—H17D	56.3
C4^ii^—C3—H3	119.4	N4—C17—H17E	109.5
C3^ii^—C4—C2	119.7 (6)	H17A—C17—H17E	56.3
C3^ii^—C4—N3	115.9 (9)	H17B—C17—H17E	141.1
C2—C4—N3	124.3 (8)	H17C—C17—H17E	56.3
C3^ii^—C4—H4	120.2	H17D—C17—H17E	109.5
C2—C4—H4	120.2	N4—C17—H17F	109.5
O4—C5—O6	120.5 (6)	H17A—C17—H17F	56.3
O4—C5—C6	120.2 (8)	H17B—C17—H17F	56.3
O6—C5—C6	119.3 (8)	H17C—C17—H17F	141.1
O4—C5—Y1	60.4 (3)	H17D—C17—H17F	109.5
O6—C5—Y1	60.1 (3)	H17E—C17—H17F	109.5
C6—C5—Y1	178.5 (5)	N4—C18—H18A	109.5
C8—C6—C7	118.5 (7)	N4—C18—H18B	109.5
C8—C6—C5	121.4 (8)	H18A—C18—H18B	109.5
C7—C6—C5	120.0 (8)	N4—C18—H18C	109.5
C6—C7—C8^iii^	120.4 (8)	H18A—C18—H18C	109.5
C6—C7—N1A	126.5 (13)	H18B—C18—H18C	109.5
C8^iii^—C7—N1A	113.1 (13)	N4—C18—H18D	109.5
C6—C7—H7	119.8	H18A—C18—H18D	141.1
C8^iii^—C7—H7	119.8	H18B—C18—H18D	56.3
C6—C8—C7^iii^	121.1 (8)	H18C—C18—H18D	56.3
C6—C8—N1	124.9 (15)	N4—C18—H18E	109.5
C7^iii^—C8—N1	111.8 (15)	H18A—C18—H18E	56.3
C6—C8—H8	119.4	H18B—C18—H18E	141.1
C7^iii^—C8—H8	119.4	H18C—C18—H18E	56.3
O7^i^—C9—O3	121.8 (6)	H18D—C18—H18E	109.5
O7^i^—C9—C10	120.3 (7)	N4—C18—H18F	109.5
O3—C9—C10	117.9 (7)	H18A—C18—H18F	56.3
O7^i^—C9—Y1^i^	46.1 (3)	H18B—C18—H18F	56.3
O3—C9—Y1^i^	75.7 (4)	H18C—C18—H18F	141.1
C10—C9—Y1^i^	166.3 (6)	H18D—C18—H18F	109.5
C12—C10—C11	117.7 (6)	H18E—C18—H18F	109.5
C12—C10—C9	121.8 (7)	C8—N1—H1A	120.0
C11—C10—C9	120.5 (7)	C8—N1—H1B	120.0
C12^iv^—C11—C10	121.1 (6)	H1A—N1—H1B	120.0
C12^iv^—C11—H11	119.5	C7—N1A—H1A1	120.0
C10—C11—H11	119.5	C7—N1A—H1A2	120.0
C11^iv^—C12—C10	121.2 (6)	H1A1—N1A—H1A2	120.0
C11^iv^—C12—N2	115.9 (8)	C12—N2—H2A	120.0
C10—C12—N2	122.9 (8)	C12—N2—H2B	120.0
C11^iv^—C12—H12	119.4	H2A—N2—H2B	120.0
C10—C12—H12	119.4	C4—N3—H3A	120.0
O2—C13—N5	136.2 (13)	C4—N3—H3B	120.0
O2—C13—H13	111.9	H3A—N3—H3B	120.0
N5—C13—H13	111.9	C3—N3A—H3A1	120.0
N5—C14—H14A	109.5	C3—N3A—H3A2	120.0
N5—C14—H14B	109.5	H3A1—N3A—H3A2	120.0
H14A—C14—H14B	109.5	C16—N4—C18	129.6 (14)
N5—C14—H14C	109.5	C16—N4—C17	118.0 (12)
H14A—C14—H14C	109.5	C18—N4—C17	112.3 (12)
H14B—C14—H14C	109.5	C13—N5—C15	121.5 (15)
N5—C14—H14D	109.5	C13—N5—C14	123.9 (14)
H14A—C14—H14D	141.1	C15—N5—C14	113.4 (13)
H14B—C14—H14D	56.3		
			
O3—Y1—O1—C1^i^	−15.5 (6)	Y1—O5—C1—O1^i^	−3.7 (11)
O5—Y1—O1—C1^i^	45.7 (6)	Y1—O5—C1—C2	176.1 (4)
O7—Y1—O1—C1^i^	111.5 (6)	O5—C1—C2—C3	166.7 (6)
O8—Y1—O1—C1^i^	−173.4 (6)	O1^i^—C1—C2—C3	−13.4 (9)
O2—Y1—O1—C1^i^	−99.3 (6)	O5—C1—C2—C4	−15.5 (9)
O6—Y1—O1—C1^i^	173.9 (5)	O1^i^—C1—C2—C4	164.4 (6)
O4—Y1—O1—C1^i^	−76.3 (6)	C4—C2—C3—C4^ii^	−0.4 (12)
C5—Y1—O1—C1^i^	−125.7 (8)	C1—C2—C3—C4^ii^	177.5 (6)
C9^i^—Y1—O1—C1^i^	90.3 (6)	C4—C2—C3—N3A	−179 (5)
O3—Y1—O2—C13	−90.5 (14)	C1—C2—C3—N3A	−1(5)
O1—Y1—O2—C13	−11.4 (14)	C3—C2—C4—C3^ii^	0.3 (12)
O5—Y1—O2—C13	−148.1 (14)	C1—C2—C4—C3^ii^	−177.5 (6)
O7—Y1—O2—C13	47.4 (15)	C3—C2—C4—N3	175.5 (10)
O8—Y1—O2—C13	67.2 (14)	C1—C2—C4—N3	−2.3 (13)
O6—Y1—O2—C13	136.8 (14)	Y1—O4—C5—O6	0.9 (7)
O4—Y1—O2—C13	−177.3 (15)	Y1—O4—C5—C6	−178.3 (6)
C5—Y1—O2—C13	159.4 (14)	Y1—O6—C5—O4	−0.9 (7)
C9^i^—Y1—O2—C13	6.9 (16)	Y1—O6—C5—C6	178.3 (6)
O3—Y1—O4—C5	157.9 (4)	O3—Y1—C5—O4	−23.3 (5)
O1—Y1—O4—C5	−143.2 (4)	O1—Y1—C5—O4	81.3 (8)
O5—Y1—O4—C5	80.1 (4)	O5—Y1—C5—O4	−91.7 (4)
O7—Y1—O4—C5	26.3 (5)	O7—Y1—C5—O4	−160.0 (4)
O8—Y1—O4—C5	−56.1 (4)	O8—Y1—C5—O4	127.0 (4)
O2—Y1—O4—C5	−119.1 (5)	O2—Y1—C5—O4	55.4 (4)
O6—Y1—O4—C5	−0.5 (4)	O6—Y1—C5—O4	179.1 (7)
C9^i^—Y1—O4—C5	57.5 (6)	C9^i^—Y1—C5—O4	−140.8 (4)
O3—Y1—O5—C1	33.5 (7)	O3—Y1—C5—O6	157.6 (4)
O1—Y1—O5—C1	−28.1 (7)	O1—Y1—C5—O6	−97.8 (7)
O7—Y1—O5—C1	−96.8 (7)	O5—Y1—C5—O6	89.2 (4)
O8—Y1—O5—C1	−142.4 (6)	O7—Y1—C5—O6	20.9 (4)
O2—Y1—O5—C1	92.6 (7)	O8—Y1—C5—O6	−52.1 (4)
O6—Y1—O5—C1	177.2 (7)	O2—Y1—C5—O6	−123.7 (4)
O4—Y1—O5—C1	120.9 (7)	O4—Y1—C5—O6	−179.1 (7)
C5—Y1—O5—C1	148.7 (7)	C9^i^—Y1—C5—O6	40.1 (5)
C9^i^—Y1—O5—C1	−74.1 (7)	C8—C6—C7—C8^iii^	−2.4 (14)
O3—Y1—O6—C5	−29.7 (5)	C5—C6—C7—C8^iii^	−177.8 (7)
O1—Y1—O6—C5	137.5 (4)	C8—C6—C7—N1A	179 (2)
O5—Y1—O6—C5	−82.2 (4)	C5—C6—C7—N1A	4(3)
O7—Y1—O6—C5	−159.9 (4)	C7—C6—C8—C7^iii^	2.4 (14)
O8—Y1—O6—C5	124.7 (4)	C5—C6—C8—C7^iii^	177.8 (7)
O2—Y1—O6—C5	56.9 (4)	C7—C6—C8—N1	164 (2)
O4—Y1—O6—C5	0.5 (4)	C5—C6—C8—N1	−20 (3)
C9^i^—Y1—O6—C5	−146.5 (4)	O7^i^—C9—C10—C12	−8.9 (10)
O3—Y1—O7—C9^i^	5.9 (5)	O3—C9—C10—C12	171.8 (6)
O1—Y1—O7—C9^i^	−63.8 (4)	Y1^i^—C9—C10—C12	−16 (2)
O5—Y1—O7—C9^i^	69.1 (4)	O7^i^—C9—C10—C11	171.4 (6)
O8—Y1—O7—C9^i^	−140.8 (5)	O3—C9—C10—C11	−8.0 (9)
O2—Y1—O7—C9^i^	−121.2 (5)	Y1^i^—C9—C10—C11	163.8 (15)
O6—Y1—O7—C9^i^	143.9 (4)	C12—C10—C11—C12^iv^	1.1 (12)
O4—Y1—O7—C9^i^	122.4 (4)	C9—C10—C11—C12^iv^	−179.2 (7)
C5—Y1—O7—C9^i^	134.4 (4)	C11—C10—C12—C11^iv^	−1.1 (12)
O3—Y1—O8—C16	−84.4 (9)	C9—C10—C12—C11^iv^	179.2 (7)
O1—Y1—O8—C16	−44.2 (8)	C11—C10—C12—N2	176.7 (11)
O5—Y1—O8—C16	88.6 (9)	C9—C10—C12—N2	−3.1 (14)
O7—Y1—O8—C16	42.4 (9)	Y1—O2—C13—N5	−162.0 (14)
O2—Y1—O8—C16	−125.5 (9)	Y1—O8—C16—N4	168.8 (9)
O6—Y1—O8—C16	129.0 (9)	O8—C16—N4—C18	−172.8 (16)
O4—Y1—O8—C16	173.0 (8)	O8—C16—N4—C17	2(2)
C5—Y1—O8—C16	151.0 (9)	O2—C13—N5—C15	−8(3)
C9^i^—Y1—O8—C16	28.2 (9)	O2—C13—N5—C14	−175.0 (19)

Symmetry codes: (i) −*x*+1, −*y*+1, −*z*+1; (ii) −*x*+1, −*y*+2, −*z*+1; (iii) −*x*+2, −*y*+2, −*z*+2; (iv) −*x*+2, −*y*+1, −*z*+1.
